# Lipid Peroxidation Products in Human Health and Disease 2014

**DOI:** 10.1155/2014/162414

**Published:** 2014-09-14

**Authors:** Kota V. Ramana, Sanjay Srivastava, Sharad S. Singhal

**Affiliations:** ^1^Department of Biochemistry and Molecular Biology, University of Texas Medical Branch, Galveston, TX 77555, USA; ^2^Environmental Cardiology, University of Louisville, Louisville, KY 40202, USA; ^3^Department of Diabetes and Metabolic Diseases Research, Beckman Research Institute, City of Hope National Medical Center, Duarte, CA 91010, USA

Lipid peroxidation is a complex chain reaction process due to the oxygen-free radicals mediated attack of cell membrane lipids such as polyunsaturated fatty acids (PUFA) resulting in cell damage and dysfunction. The end products of lipid peroxidation yield a variety of highly reactive electrophilic aldehydes, which can act as endogenous danger signals that alter important cell signaling pathways responsible for disease pathogenesis. Recent studies identified potential role of lipid peroxidation products as markers of oxidative stress and biomarkers of human diseases. Indeed, a number of preclinical and clinical studies suggest the involvement of lipid peroxidation products in numerous pathological conditions such as inflammation, atherosclerosis, diabetes, ageing, neurodegenerative diseases, and cancer. The wealth of knowledge we are gathering from the past decade or so will significantly help us to better understand the mechanisms by which lipid peroxidation products trigger pathological aspects and will help to identify novel potential targets for future therapeutic strategies.

The 2014 special issue of lipid peroxidation products in human health and disease compiles 16 excellent manuscripts, including clinical studies, research articles and reviews, which provides comprehensive evidence demonstrating the significance of lipid peroxidation products in various pathological conditions.

The 3 review articles of this issue discuss how lipid peroxidation products are involved in cell signaling which leads to various pathological conditions. An excellent review article by A. Ayala et al. described in depth how lipid peroxidation-derived aldehydes such as malondialdehyde (MDA) and 4-hydroxynonenal (HNE) are formed from PUFA and their metabolism. Further, they described how MDA and HNE are involved in cell signaling that causes either cell survival or cell death. At the end, they also discussed various* in vivo* mammalian model systems used for investigating lipid peroxidation. The review article by E. Miller et al. described the significance of isoprostanes and neuroprostanes as biomarkers of oxidative stress in various neurodegenerative diseases. Specifically, authors have nicely discussed the relationship between F2-isoprostanes and F4-neuroprostanes as biomarkers of lipid peroxidation in the pathogenesis of human neurodegenerative diseases such as multiple sclerosis, Alzheimer's disease, Huntington's disease, and Creutzfeldt-Jakob disease. Joshi and Pratico in their review article discussed how lipid peroxidation is involved in the pathophysiology of psychiatric diseases. Specifically, the authors have nicely described recent clinical data supporting the involvement of lipid peroxidation aldehydes in schizophrenia, bipolar, and other major depressive disorders. The review articles in this special issue provide widespread information on the formation of lipid peroxidation-derived aldehydes and their involvement in the neurological and psychiatric diseases.

The research article by M. E. Soto et al. investigates the role of oxidative stress in various aortopathies. In this study, aorta fragments from patients with systemic arterial hypertension, Marfan's syndrome, Turner's syndrome and Takayasu's arteritis were evaluated for oxidative stress enzymes and structural and functional proteins. This study indicates that the activities of glutathione peroxidase and glutathione-S-transferase were decreased and the rate of lipid peroxidation was increased in all types aortopathies. However, activities of other oxidative and functional proteins showed variations depending upon the type of aortopathy. A cross-sectional study by F. Moreto et al. examines the relationship between plasma lipid peroxidation product MDA and metabolic syndrome in 148 free-living human subjects. Authors found interesting data that subjects with higher plasma MDA showed higher prevalence of metabolic syndrome accompanied by higher waist circumference, higher values of glucose, triglycerides, insulin resistance, and higher dietary sugar-intake. These studies indicate that MDA is a major determinant of glucolipotoxic state in subjects with metabolic syndrome.

Studies by P. Stiuso et al. report oxidant/antioxidant status and lipidomic profile in the serum of NASH patients at the basal conditions and after one-year treatment with the silibinin-based food integrator Realsil, they found that chronic treatment with Realsil significantly changed the basal severity of the disease as determined by NAS scores and most importantly decreased the serum lipid peroxidation. These data indicate that lipidomic status in the serum of patients with NASH could be a useful prognostic marker for the antioxidant therapies. In another study by S. Dziegielewska-Gesiak et al. investigated the role of lipid peroxidation products, plasma total antioxidant status and Cu-, Zn-SOD as biomarkers of oxidative stress in elderly prediabetic subjects. Based on the data obtained from 52 elderly persons, this study identified SOD-1 and TAS as initial stage biomarkers and thiobarbituric acid-reacting substances (TBARS) as later stage biomarkers of oxidative stress in the elderly prediabetics.

Another study by P. Sutkowy et al. examined the influence of exercise combined with whole-body cryotherapy on lipid peroxidation products formation in healthy kayakers. This study reports data on various oxidative stress and antioxidative markers such as MDA, conjugated dienes, protein carbonyls, total antioxidant capacity, vitamin E, cortisol, and testosterone in 16 kayakers of the Polish National Team. The findings indicate that combining exercise during longer training cycles with whole-body cryotherapy could be advantageous in kayakers. A multicentric research study reported by L. M. Gomez-Olivan et al. examined the impact of involuntary exposure of antineoplastic drugs in nurses in Mexican hospitals. When compared to occupationally exposed nurses with control subjects who are not occupationally exposed to antineoplastic drugs, the occupationally exposed nurses show significantly increased levels of oxidative stress markers, including lipid peroxidation levels, protein carbonyl content, super oxide dismutase, catalase, and glutathione peroxidase. This study again addresses the significance of lipid peroxidation products as biomarkers of oxidative stress in antineoplastic exposed subjects.

M. Saenz-de-Viteri et al. created an interesting model to investigate phototoxicity in the rabbits exposed to light. By using this model, they found that retinas from rabbits exposed to light showed higher levels of lipid peroxidation than unexposed controls. They demonstrate that light damage causes an increase in the retinal oxidative stress immediately after light exposure that decreases with time of exposure, although, some morphological and apoptotic events still appear days after light exposure. Buttari et al. investigated the effect of polyphenolic compound resveratrol to regulate the 7-oxo-cholesterol-triggered proinflammatory signaling in M1 and M2 human macrophage subsets. These studies demonstrate that in the M1 subset, resveratrol prevents the 7-oxo-choesterol-induced downregulation of CD16 and the upregulation of MMP-2 extracts whereas in M2 subset, resveratrol prevents the upregulation of CD14, MMP-2, MMP-9, and downregulation of endocytosis. These studies also indicate that regulation of NF-*k*B activation is the main signaling mechanism by which 7-oxo-choesterol and resveratrol mediate inflammatory effects.

S. A. Ganie et al. in their article reported the antioxidant and cytotoxic activities of* Arnebia benthamii*, an endangered medicinal plant of Kashmir Valley. By using rat liver microsomes and human cancer cell lines, authors have shown the antioxidant potential of this plant extract. Specifically, they have shown that extract of Arnebia inhibits Fe2+/ascorbic acid-induced lipid peroxidation in rat liver microsomes and also shows cytotoxic effects towards various cancer cell lines. The effect of antihypertensive drug Enalapril on oxidative stress markers and antioxidant enzymes in the kidneys of spontaneously hypertensive rats was reported by G. Chandran et al. in their article. Particularly, they demonstrate that Enalapril treatment significantly enhanced total antioxidant status and superoxide dismutase and decreased the TBARS levels in the kidneys of hypertensive rats. These studies indicate that Enalapril, besides its antihypertensive effect, also decreases the oxidative stress and lipid peroxidation in the hypertensive rat kidneys. In another research article by S. Ojha et al. reported the effect of* Withania coagulans* fruit extract on oxidative stress and inflammatory response in the kidneys of streptozotocin-induced diabetic rats. They have shown that treatment of diabetic rats with* Withania* plant extract significantly decreased hyperglycemia, glutathione levels, inflammatory cytokines such as IL-1b, IL-6, and TNF-α, and subsequent renal injury. These studies indicate potential antioxidative and anti-inflammatory actions of this fruit extract in prevention of diabetic complications.

S. Perrone et al. in their clinical study investigated the hypothesis that neonatal supplementation of lutein in the first hours of life reduces neonatal oxidative stress in the immediate postpartum period. This hypothesis was tested in a randomized, double-blinded clinical trial conducted among 150 newborns by investigating the levels of total hydroperoxides, advanced oxidation protein products, and biological antioxidant potential in the blood samples collected from the cord. Their findings indicate that neonatal supplementation of lutein in the first hours of life increases biological antioxidant potential and reduce total hydroperoxides when compared to babies without lutein supplementation. In another interesting randomized cross-over clinical study, L. D. Renzo et al. evaluated the outcome of consumption of McDonald's meal and a Mediterranean meal without and with red wine in healthy population. Red wine decreased the ox-LDL and increased the expression of antioxidant enzymes in people taking McDonald's or Mediterranean meals. These studies indicate the positive effect of red wine intake combined with widely consumed meal types on oxidative and inflammatory gene expressions.

As an end note, it is obvious from the recently published studies and current special issue papers that lipid peroxidation products play a major role in human health and disease. Lipid peroxidation products are now recognized as biomarkers of oxidative stress and endogenous danger mediators of multiple cell signaling pathways. Although, over the past several years, substantial research has shown that lipid peroxidation has a crucial pathophysiological role in the development of various human diseases, the exact nature of the significance of lipid peroxidation on cellular homeostasis that maintain cell survival, differentiation, and death leading to pathological consequences and their responses to antioxidant therapies requires further detailed investigations. Newly developed technologies such as lipid finger printing/lipidomics and metabolomics are important tools that will help to define how the lipid peroxidation products adapt and provide a buffer against increased oxidative stress in various pathological conditions. Therefore, the molecules that interrupt or neutralize the effects of lipid peroxidation products-mediated signaling pathways could be the next important targets for future drug discovery studies.

